# How to Manage a Patient with Ocular Metastases?

**DOI:** 10.3390/biomedicines10123044

**Published:** 2022-11-25

**Authors:** Juliette Thariat, Laurys Boudin, Olivier Loria, Anh-Minh Nguyen, Laurent Kodjikian, Thibaud Mathis

**Affiliations:** 1 Laboratoire de Physique Corpusculaire IN2P3-CNRS UMR 6534 ARCHADE, Unicaen Université de Normandie, 6 bd du Maréchal Juin, 14050 Caen, France; 2 Service d’Oncologie, Hôpital d’Instruction des Armées Sainte Anne, 2 bd Sainte-Anne BP600, 83000 Toulon, France; 3 Service d’Ophtalmologie, Hôpital Universitaire de la Croix-Rousse, Hospices Civils de Lyon, 103 Grand-rue de la Croix-Rousse, 69317 Lyon, France; 4 Laboratoire UMR5510 MATEIS, CNRS, INSA Lyon, Université Lyon 1, 345 av. Gaston Berger, 69100 Villeurbanne, France

**Keywords:** plaque brachytherapy, choroidal metastasis, ocular tumor, proton therapy, radiotherapy

## Abstract

Ocular metastases are the most frequent ocular malignant tumors; their prevalence is estimated around 5–10% and is even higher in patients with breast or lung cancer. They represent various clinical situations, but they share the same hierarchical multidisciplinary therapeutic challenge with respect to the way systemic and local therapies should be selected in combination or sequentially in the personalized medical history of a patient. The challenges include tumor control, eye preservation, and the minimization of iatrogenic damage to sensitive tissues surrounding the tumor in order to preserve vision. These aims should further contribute to maintaining quality of life in patients with metastases. Many patients with choroidal metastases have systemic molecular treatment for their primary tumor. However, secondary resistance to systemic treatment is common and may ultimately be associated with cancer relapse, even after an initial response. Therefore, it makes sense to propose local treatment concomitantly or after systemic therapy to provide a more sustainable response. The aim of this review is to present current therapeutic strategies in ocular metastases and discuss how to tailor the treatment to a specific patient.

## 1. Introduction

Ocular metastases are the most frequent ocular malignant tumors and are probably underdiagnosed in the context of metastatic cancers. Their detection has dramatically increased since advances in diagnostic tools and with improved survival of patients with metastases [1,2]. The management of patients with metastatic cancer is complex and multidisciplinary as it should not only aim at prolonging survival but also at preserving quality of life. Accordingly, life-threatening or painful metastases require rapid treatment and are among the highest priorities. There are many other situations where patients with metastases will benefit from specific anti-cancer treatments and symptomatic treatments.

From epidemiological and post-mortem studies in cancer patients, the prevalence of ocular metastases is estimated to be around 5–10% and is even higher in case of breast or lung cancer [3]. Although ocular metastases are not immediately life-threatening, vision loss and blindness significantly alter the quality of life, reduce autonomy, and are among the leading causes of fear in patients. Therefore, ocular metastases should be taken into account regardless if they are isolated, or associated with multiple visceral/bone metastases hampering the patient’s overall prognosis [4]. In the metastatic context, however, the ocular examination is generally omitted even in the presence of mild to moderate ocular symptoms. Yet, the methods of detection of ocular metastasis have improved and use imaging techniques that are common in ophthalmology practice: fundoscopy, fluorescein and indocyanine green angiography, optical coherence tomography (OCT), and ultrasonography (US, including ultrasound biomicroscopy–UBM). Ocular metastases can be detected clinically in the case of an iris metastasis, or on a simple fundoscopy examination for retinal and choroidal metastases. A more comprehensive ophthalmological examination can confirm the diagnosis, the exact localization in ocular tissue, the number (and focal or diffuse pattern) and uni- or bi-laterality of the ocular mass, and assess the visual and ocular consequences of the tumor [5,6]. 

Ocular metastases represent various clinical situations, but they share the same hierarchical multidisciplinary therapeutic challenge: whether to deliver systemic and/or local treatment, simultaneously or sequentially. The objectives include tumor control, eye preservation, and the minimization of iatrogenic damage to sensitive tissues surrounding the tumor to preserve vision. These aims should further contribute to maintaining quality of life in patients with metastases.

The goal of this review is to present current therapeutic strategies in ocular metastases and discuss how to tailor the treatment to a specific patient.

## 2. Methods

This article is based on a review of the literature and consensus among ocular oncologist specialists, including ophthalmologists, medical oncologists, and radiation therapists. A literature search was performed in July 2022 using PubMed to identify relevant publications related to ocular metastases. The aim of this review–expert opinion is to describe the different treatment options for ocular metastases and to discuss specific management based on disease characteristics and localization.

## 3. Ocular Metastasis Localization

**Figure 1:**
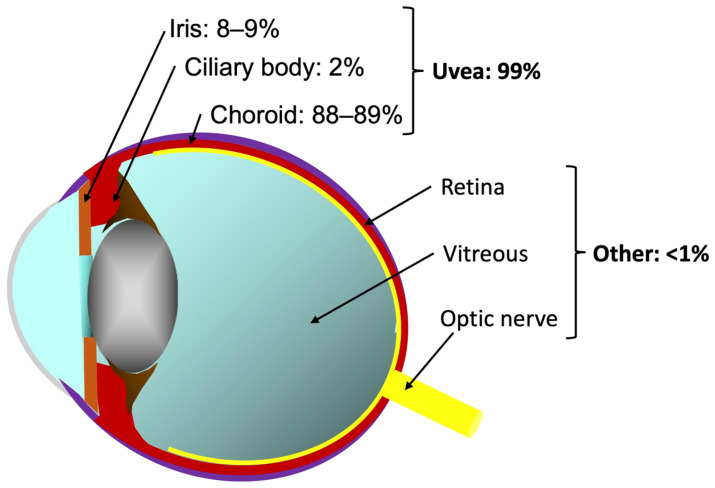
Ocular metastasis localization.

The ocular compartment is richly vascularized but is well-known as a «sanctuary» because the blood-aqueous and the blood-retinal barriers prevent the passage of cells and drugs into the intra-ocular compartment. The choroid is the only intraocular tissue that is located outside these barriers, explaining why the vast majority of ocular metastases are located in this compartment (almost 90%) [3,7]. On the contrary, metastases to the retina, optic disc and vitreous are rare, and only a few observations are described in the literature [3,8,9]. Metastasis to the iris is also an uncommon situation (about 8–9% of cases) [10]. The iris, just like the choroid, is part of the uvea, but the abundant muscular tissue and the reduced blood flow in the iris account for this difference in incidence (Figure 1).

Except for vitreous metastasis, which are revealed by the presence of vitreous cells, ocular metastases are usually seen as achromatic, hypervascular masses. If pigmentation is present, it can hint at specific primary tumors (e.g., brown for skin melanoma, orange for renal or thyroid metastases). Multimodal imaging, including slit lamp examination, fundoscopy, US, OCT, fluorescein and indocyanine green angiography, helps to determine the exact localization, numbers, diameter, and thickness of each metastasis. These lesions can compromise vision directly because of their localization, but also because of the complications they trigger. For example, retinal and choroidal metastases with hypervascularization and increased vascular permeability can induce retinal detachment. Iris and ciliary body metastases can induce trabecular obstruction leading to ocular hypertension and glaucoma.

## 4. Epidemiology and Clinical Features of Ocular Metastases

Metastatic tumors are the most common ocular malignancy, although they are relatively underdiagnosed in patients with other symptoms related to their primary cancer and other life-threatening metastases. This explains why post-mortem histopathological studies on patients who died from cancer revealed a higher prevalence of ocular metastases than that reported in living cancer patients [11,12,13,14,15,16,17].

The most frequent primary neoplasms at the origin of ocular metastases are breast and lung cancers, which account for almost 75% of cases [3,7,18]. Other localizations include gastrointestinal tract, prostate, kidney, skin…. In a patient with no history of cancer, at least lung and breast investigations should be done after ocular metastasis diagnosis. Moreover, patient and tumor characteristics may hint to a type of cancer. For example, ocular metastases from breast cancer are usually multifocal and bilateral and associated with concurrent systemic metastases [3,7,19,20]. In comparison, ocular metastasis from lung cancer precedes the diagnosis of the primary in about 50% of cases and generally affects men [19,20].

## 5. Treatment Paradigm

Once cancer has spread to the eye, the prognosis of patients is significantly altered [8,20]. Yet there is important variability of prognosis between primary tumors, and between oligo and multimetastatic diseases [5]. The choroidal localization is the most frequently encountered site of ocular spread of metastatic cancers. Metastases located in other ocular tissues (iris, retina, vitreous and optic disc) are often detected in end-of-life in a multimetastatic context, after patients have received several lines of systemic treatments, or when they are in palliative care [3,8,9]. Therefore, these metastases are commonly undertreated. 

Because ocular metastases are non-life-threatening, the goal of the treatment is to preserve vision. This can be achieved by decreasing the tumor volume and by the management of induced ocular complications. The treatment outcome can thus be monitored by the decrease in tumor volume (assessed clinically or by ultrasonography), the decrease in tumor-related exudation (i.e., exudative retinal detachment visualized clinically or by OCT), and ultimately by the stability or improvement in vision (assessed by visual acuity testing). It should also be noted that uncontrolled progression of an ocular metastasis can lead to phthisis bulbi and ocular pain, sometimes compromising globe anatomy [3,10]. Regular ophthalmologic assessment is therefore necessary and should at least include visual acuity testing, slit lamp examination/fundoscopy, US and OCT (in case of posterior segment metastasis). Other imaging modalities such as angiography, can be discussed on a case-by-case basis.

Altogether, the treatment of ocular metastases should be easy to implement without interfering with the systemic cancer treatment. It should not delay the treatment of extra-ocular metastatic sites and should not alter the patient’s quality of life. The treatment of ocular metastases should also be effective in improving vision and be associated with a low risk of complications.

Local treatments, using conventional radiotherapy or non-invasive methods such as photodynamic therapy (PDT) have been quite popular. However, recent advances in systemic therapies have significantly improved the local control rates, and sometimes survival, of patients with metastases. Subsequently, it has been assumed that ocular tumor response under systemic therapy would remove the need for local treatments. It is true that some patients may benefit from an effective and durable ocular treatment that will preserve vision throughout their prolonged survival, which can sometimes reach several years [21]. However, in the numerous non-responders or those who respond in several metastatic sites but not in their ocular metastasis, postponing the local treatment may lead to blindness. Therefore, the sequence and timing of the local treatment is critical. 

Moreover, most patients with ocular metastases have systemic therapies for their primary tumor or metastatic spread [19,20,22,23]. Secondary resistance to classical systemic chemotherapies is common and may ultimately be associated with treatment failure [24,25]. In a series of 194 patients with lung cancer and choroidal metastases, Shah et al. reported that ocular radiotherapy in addition to systemic therapy decreased the rate of relapse significantly and provided higher tumor response rates than systemic therapy alone [20]. However, little is known about the efficacy of targeted therapy or immunotherapy on choroidal metastasis. Overall, a concomitant local treatment could be proposed initially or shortly after systemic therapy to ensure a long-term response. The choice of technique depends on several factors including the number of ocular metastases, their localization, visual prognosis, and life expectancy.

## 6. Treatment Options

The different treatments available for ocular metastases are presented below and in Table 1. We mainly develop treatment options for choroidal metastases because they are the most frequent. There is poor literature data on treatment options for metastasis to the retina, vitreous, iris and ciliary body. 

### 6.1. Systemic Treatments

The choroid is a highly vascularized tissue composed of large choroidal vessels and fenestrated choriocapillaris endothelium, explaining why ocular metastases frequently lie in the choroidal compartment. This richly vascularized and permeable environment allows high penetration of anti-cancer molecules from the blood into the choroidal compartment [26].

#### 6.1.1. Immunotherapy

Specific aspects of choroidal microenvironment participate in the physiological mechanisms that the eye uses to give tumor metastases a growth advantage as an immunologically privileged organ [27]. A recent study showed that this immune-privileged microenvironment limited the effectiveness of immunotherapy in an intraocular metastasis mouse model. The non-tumoral choroid tissue further showed high Fas-ligand and PD-L1 expression, and low CD8+ T-cell infiltration, which seems to strongly inhibit tumor response under immunotherapy. The mechanism by which immunotherapy induces tumor response may be different in the eye than in other tumor sites [28]. There are very few clinical data reporting the effectiveness of immunotherapy on ocular metastases in the literature. In a recent case report, a favorable response of choroidal metastases was described in a non-small cell lung cancer with checkpoint inhibitor pembrolizumab, in association with pemetrexed and capecitabine [29]. In addition, responses of extra-ocular metastatic sites to immunotherapy are often limited. Among cancers most frequently associated with choroidal metastases, immunotherapy has demonstrated overall survival benefits in triple-negative breast cancer, lung cancer and kidney cancer [30,31,32]. When combined with chemotherapy in breast or non-small cell lung cancer, response rates can reach approximately 50% [30,33]. For other tumors, the estimated percentage of patients eligible for immune checkpoint inhibitors in the United States was around 44% in 2018 with an estimated response rate of 13% [34]. Although response rates with immunotherapy can reach 58% for first-line metastatic melanomas (“hot” tumors) [35], for most cancers, immunotherapy alone does not allow a decrease in tumor size [33]. Therefore, the addition of local treatment to an immune checkpoint inhibitor should be strongly considered in cases of choroidal metastasis, especially in cold tumors (which have a low mutational burden and immune scores using histological/immunohistochemistry biomarkers). It should also be noted that response may be delayed with immunotherapy.

#### 6.1.2. Chemotherapy

Concerning chemotherapy, numerous studies have reported its effectiveness in addition to local irradiation in the management of choroidal metastases, but very few have specifically studied its effectiveness alone. Yang et al. evaluated 4 patients with choroidal metastasis of non-squamous cell carcinoma treated with pemetrexed and cisplatin. They observed 3 cases of response under chemotherapy alone but there was no follow-up after the initial response [23]. Shah et al. evaluated the clinical features, treatment, and prognosis of 374 uveal metastases from lung cancer including 88%, 2% and 10% of choroid, ciliary body, and iris metastases respectively. Chemotherapy alone was administered to only 22 patients with 68% of tumor regression and 32% of progression. In this large study, chemotherapy was associated with local treatment for most patients, illustrating that durable tumor control is generally achieved by ocular irradiation [20]. Therefore, chemotherapy may be attempted in case of rapidly progressing or multivisceral disease, but the response should be monitored early so as to switch to local therapy in case of poor ocular tumor response.

#### 6.1.3. Targeted Therapy

Depending on cancer specific mutations, targeted molecules can also be used. Treatment may be personalized on the basis of biomarkers present in the patient’s tumor tissue to increase sensitivity to treatment. Target therapies may also be chosen for their different patterns of systemic adverse events, compared to chemotherapy. This is particularly the case for lung and breast cancers. Maller et al. conducted a systematic review of intraocular metastasis from lung cancer in the era of targeted therapy. In their study, 45% of patients had targeted therapy with a median overall survival of 27 months and 92% had stable or improved vision. The use of targeted therapy was the strongest predictor of improved survival, and ocular radiotherapy was also associated with better outcome [36].

For breast cancer expressing estrogen or progesterone receptors, hormonotherapy can be considered a targeted therapy. Interestingly, this breast cancer subtype is associated with more choroidal metastases but these choroidal metastases show favorable ocular response to hormonal therapy [5,37]. CDK 4–6 inhibitors recently improved the prognosis of breast cancer in combination with hormone therapy with a clinical benefit rate of 80% [38]. This therapeutic class also seemed effective on choroidal metastases [39]. 

For HER2-positive breast cancers, new targeted therapies effective on central nervous system (CNS) metastases are now available. Tucatinib, a small-molecule HER2 kinase inhibitor with the potential to penetrate the blood brain barrier, demonstrated a median CNS-progression free survival of 9.5 months [40]. Trastuzumab deruxtecan, an antibody-drug conjugate, yielded a dramatic intracranial response rate of 73% in second-line setting [41]. These data suggest the potential efficacy of some targeted therapies on ocular metastases in hormonal-positive breast cancer. More and more published case reports have shown durable improvement of vision under targeted therapy alone and question the systematic use of local therapy when a good tumor response is obtained [5]. However, case reports are biased and only those showing positive results are likely to be published. 

#### 6.1.4. Opinion on Systemic Treatment

There are few data on the use of systemic treatments without local treatment for choroidal metastases in the literature. Nevertheless, choroidal vascularization seems to allow the diffusion of anti-cancer treatments. Chemotherapy and mainly targeted therapies frequently achieve tumor response. The particular microenvironment of the choroid potentially impairs the efficacy of immune checkpoint inhibitors. The absence of clinical data concerning immunotherapy alone in this situation makes its routine use uncertain. We believe that for choroidal metastases, the administration of systemic treatment, especially targeted therapy, is essential as it has demonstrated its efficacy in metastatic situations. As with any metastatic location, local treatment is also required in the event of symptoms. However, given the lack of data on systemic treatments with immunotherapy and the functional risk in the event of progression, radiotherapy should always be discussed. It is important to note that apart from breast cancer with HER2 amplification, the response rates of cancers in second-line therapy rarely exceed 20% [42,43,44]. This low second-line response rate mandates a multidisciplinary discussion as soon as choroidal metastasis is diagnosed. Because the anti-tumor response is not predictable, and choroidal metastasis will become too large and result in permanent ocular damage under inefficient systemic treatments, it is important to anticipate the use of local treatments and to limit the number of lines and cycles of chemotherapy before advocating for ocular radiotherapy or other effective local treatments. The survival benefit associated with radiotherapy in patients with choroidal metastases of lung cancer highlights the importance of this treatment [36].

### 6.2. Local Treatment Techniques

Local treatment is nowadays considered the treatment of choice for choroidal metastases. It induces direct action on the tumor, by promoting cellular death, vascular thrombosis, and local inflammatory reaction inducing autophagy [45,46]. Some treatments are performed at the ophthalmologist office. Ocular radiotherapy is performed at a hospital or clinic and patient compliance is necessary to attend several radiotherapy sessions. 

#### 6.2.1. Conventional Radiotherapy

Conventional external beam radiotherapy (EBRT) using 6 MV photons is currently the standard radiation technique for the treatment of choroidal metastases. Its therapeutic ratio is relatively good, providing tumor control (response or stability) in about 90% of cases and good tolerance in most patients with metastases. Tumor response is however not immediate; it is generally delayed until 3 months after EBRT.

In a series of more than 200 patients, treated for ocular metastases, most patients (72%) received total doses of 30–40 Gy in 2–3 Gy fractions corresponding to a biologically effective dose of 50–70 Gy [47]. Higher doses may be prescribed [19]. Additionally, a normofractionated scheme is used in patients with a life expectancy greater than one year, in order to minimize the long-term side effects. However, the dose to the macula should be kept under 45 Gy in order to restore or spare vision. Short hypofractionated palliative courses are often preferred for patients with short life expectancy; the most common regimens are 30 Gy in 10 fractions, or 20 Gy in 5 fractions. The dose necessary to control choroidal metastases is therefore in the same range as the dose used to treat extra-ocular metastases. It is usually effective despite doses being lower than those used for primary tumors.

In other studies, investigating EBRT, patients were generally treated after a few cycles of systemic chemotherapy, but no control groups were included, either with chemotherapy alone or radiotherapy alone. Median follow-up rarely exceeded 1 year, probably due to the limited survival of patients with metastases. The most common dose given to the choroidal metastasis was a classical hypofractionated scheme (i.e., in a few relatively high dose fractions) of 30 Gy in 10 fractions, although shorter hypofractionation has been described with even fewer fractions, lightening the therapeutic load on end-of-life patients [19,48,49]. 

#### 6.2.2. Plaque Brachytherapy

Few studies have evaluated plaque brachytherapy in the case of choroidal metastasis with a high effectiveness and few side effects. The most frequently used radioisotope is ^125^I with a therapeutic dose of 45–70 Gy delivered at the tumor apex in 3–4 days. Tumor response is generally evaluated at 3 months after treatment, showing tumor flattening and resorption of exudative signs [50]. Several limitations prevent the use of plaques for the treatment of choroidal metastases. Unlike choroidal melanoma, choroidal metastasis growth pattern is generally flat with a large infiltration of the choroid resulting in an irregular shape [51]. Lateral margins are thus poorly defined and choroidal mass is typically multilobular with an irregular surface, making it difficult to calculate dose distribution at the apex. Secondly, most choroidal metastases are posterior with 40% reported in the macular region, a localization that is technically difficult to reach during plaque placement surgery [3,7]. 

#### 6.2.3. Proton Therapy

Proton therapy (PT) is not a standard treatment for choroidal metastasis; it is rather indicated for diseases that can be treated with curative intent. Ocular PT also requires clip placement when advocated in the treatment of uveal melanomas. However, anecdotal experience with PT is worth mentioning for selecting patients who have relatively resistant tumors and an excellent overall prognosis.

PT for choroidal metastasis treatment was evaluated in only two studies [52,53], as well as a few cases reports [54]. Tsina et al. reported 63 patients with choroidal metastases treated by ocular PT with 14 Gy relative biological effectiveness (RBE) in 2 fractions, with excellent tumor response in up to 84% of cases, and no recurrence during the mean 10 months follow-up [53]. Similarly, the cohort of 77 patients treated by Kamran et al. had 94% local control with mild-to-moderate adverse events [52]. Even if the total dose given to the tumor is lower than the dose-at-risk for the retina and optic disc, radiation-induced maculopathy and RION were sometimes reported. To prevent such side effects, hypofractionated PT using fiducials (to ensure a correct positioning of the eye and customized dose to the macula) can achieve similar outcomes and provide better macular sparing. However, PT is not a standard treatment for ocular metastases and should be discussed on a case-by-case basis. 

#### 6.2.4. Stereotactic Body Radiotherapy

Stereotactic body radiotherapy (SBRT) uses photons (gamma or X-rays) just like conventional radiotherapy. It is increasingly used in eye tumors, with a hypofractionated scheme. There are very few case series on its use for the treatment of choroidal metastases [55,56,57,58]. Published data indicate that a dose of 12–25 Gy is sufficient to effectively treat choroidal metastasis and encouraging results have been found for CyberKnife^®^ (Accuray Inc., Synnyvale, CA, USA) and GammaKnife^®^ (Elekta Solutions AB, Stockholm, sweden) SBRT. It is also possible to treat simultaneously synchronous cerebral metastases in the same session [56].

Several limitations of SBRT should be considered. The technique requires accurate fixation during treatment planning and treatment, lasting approximately 30 min. Therefore, it may not be suitable for patients with severe visual loss in both eyes as it could lead to lack of accuracy and diffuse dose distribution. There are techniques available to achieve eye immobilization, such as anesthetic ocular blocks and bridle suture, but they require invasive procedures, which may not be fully relevant to patients with metastases [56]. This may however be discussed on a case-by-case basis.

#### 6.2.5. Ophthalmological Treatments

When patients with metastases have a short-life expectancy, radiotherapy techniques are generally not indicated, and minimally invasive treatments are generally preferred. “Office-based” treatments can be performed directly by ophthalmologists and require few visits. PDT is a technique that uses verteporfin, a light-sensitive compound administered intravenously and generally used in ocular oncology for the treatment of circumscribed choroidal hemangioma [59]. Shortly after the injection, a 689-nm laser is targeted to the fundus lesion to activate verteporfin molecules, producing reactive oxygen species (ROS) which induce a cytotoxic effect and lead to thrombosis in the tissue. Several authors have reported encouraging short-term outcomes in patients with metastases with small solitary choroidal metastasis treated by PDT [60,61]. However, some failures have been described in cases of large and thick tumors [62], or aggressive multimetastatic disease [60]. PDT should be proposed for retro-equatorial choroidal metastases when they have a thickness < 3 mm and a diameter < 10 mm, since the laser will not penetrate in thicker tumors. PDT should also be avoided in case of important exudative syndrome for the same reasons [5].

The Intravitreal injections of ani-angiogenic agents such as intravitreal anti-VEGF can also be proposed, especially when the tumor does not fit the PDT criteria. Tumor cells secrete pro-angiogenic factors leading to pathological angiogenesis necessary for proliferation [63]. Choroidal metastases show hypervascularity and seem strongly dependent on VEGF and angiogenesis. Anti-VEGF injection aim to decrease this angiogenic stimuli by antagonizing VEGF, the leading angiogenic agent [64]. This assumption was confirmed when regression of choroidal metastases and functional improvement was observed following anti-VEGF injection [65,66]. Most importantly, and unlike PDT, this treatment can be given in cases of large or highly exudative tumors. However, anti-VEGF molecules have limited intravitreous half-life and frequent monitoring should be done to ensure early detection of tumor progression.

The overall clinical effectiveness of ophthalmological treatments is promising, especially for patients with short life expectancy. While radiotherapy techniques take several weeks to plan and treat the tumor, PDT and intravitreal injections can be initiated and completed in a single day without surgical procedure, improving quality of life and minimizing ocular toxicities.

**Table 1:** Pros and Cons of each treatment modality.

Treatment Modality	Pros	Cons	Conclusion
**Systemic treatment**	**May be initiated quickly and can control extraocular disease.**	**Ocular effects insufficiently reported.** **Duration of response unclear, requiring frequent monitoring.**	**May be more efficient in choroid that iris/retinal metastases due to vascular supply.**
Chemotherapy [20]	-Chemotherapy alone (22/347 patients): 68% tumor regression. -Should be attempted in case of rapidly progressing or multimetastatic disease.	-Chemotherapy is generally associated with ocular treatment: durable control by ocular irradiation. -Publication bias in favor of good response to chemotherapy alone. -Usually requires hospitalization.	A short course may be proposed with early local treatment in case of progression or poor response.
Targeted therapy [22,36]	Personalized treatment based on biomarkers (breast, lung cancers) allows individual efficient treatment (response reach up to 70%). >90% stable/improved vision in a median overall survival > 2 years.	-Better response in case of biomarker expression. -Secondary resistance may occur.	-Can be used alone as first line, but progression should rapidly prompt local treatment. -Effectiveness depends on the presence of specific biomarkers.
Immunotherapy [30,33,34,35]	-Well tolerated and some efficacy combined to chemotherapy in breast/lung cancer. -Response rates ~50%, or above in hot tumors (melanomas).	-Limited response in cold tumors: half of cancer patients, ≤15% response rates. -Delayed response.	Interesting response rates in hot tumors, should be combined in other cases.
**Local treatment**	**Good tolerance, tumor response and consistent effect on vision.**	**Ocular effects only.**	**May be used sequentially.**
Radiotherapy [19,20,47,48,49,52,53,54,55,56]	-Non-invasive treatment can be combined with systemic treatments. -Number of fractions and technique can be customized based on patient/tumor presentation and resistance.	-Fractionated treatment course -Reirradiation at risk of toxicities.	-Durable ocular tumor response. -Feasible also in case of metastasis of ocular/extraocular site.
PDT [60,61]	-Single shot treatment, may be repeated based on response monitoring. -Non-invasive (intravenous injection): can be performed in poor performance status patients.	Less efficient in case of bulky or progressing tumors.	Could be a first-hand local treatment.
Intravitreal anti-VEGF [65,66]	-Few injections: may be repeated based on response monitoring. -Minimally invasive: can be performed in poor performance status patients.	-Effect on large tumors to be demonstrated. -Requires frequent monitoring.	Duration of response to be ascertained.

PDT: photodynamic therapy; VEGF: vascular endothelial growth factor.

## 7. Which Therapy for Which Choroidal Metastasis?

Numerous questions should be raised before managing a patient with choroidal metastases: Is the choroidal metastasis responsible for ocular symptoms? What is the patient’s life expectancy? Are there other metastases and how should multimodal treatment be managed?

Many patients with choroidal metastases have systemic molecular treatment for their primary tumor. However, resistance to systemic treatment is common and may ultimately be associated with cancer relapse, even after an initial (but transient) response. Therefore, it is important to identify the optimal timing of local treatment. It can be done concomitantly or after systemic therapy and should provide a more sustainable response that will also allow better vision preservation than systemic treatment alone. Depending on the answers given to the questions above, different strategies can be proposed to the patient.

Estimation of life-expectancy of a cancer patient is challenging and is based on several parameters: the type of cancer, the stage at the time of diagnosis, the particular traits of the tumor (cell types, oncogenic mutations, …), previous treatment received and respective response, as well as physical and psychological health. However, each person responds to treatment differently and no one knows for sure how long a patient with metastases will live with cancer. If the patient’s estimated survival is short, it is best to avoid complex and time-consuming treatments, which increase the burden on the patient and the time spent away from his/her home and family. Nevertheless, palliative care aims to improve quality of life, and should include prevention of vision loss and ocular pain. 

Systemic therapy is the mainstay of metastatic cancer and patients with choroidal metastases are generally already treated by anti-cancer drugs at the time of ocular diagnosis, or soon after. Patients with specific oncogenic driver mutations or translations may exhibit dramatic tumor response under specific targeted therapies, including a decrease in tumor mass and exudative syndrome [19,20,22,23]. For these patients, secondary resistance to these molecules are described and can be associated with treatment failure [24,25]. For such patients, systemic therapy should be first evaluated, and local therapy should be considered only in case of partial or transient response associated with visual impairment. In such cases, according to the number and laterality of choroidal metastases, short and easy to implement treatment can be proposed including anti-VEGF injections, PDT, and short hypofractionated conventional EBRT.

**Figure 2:**
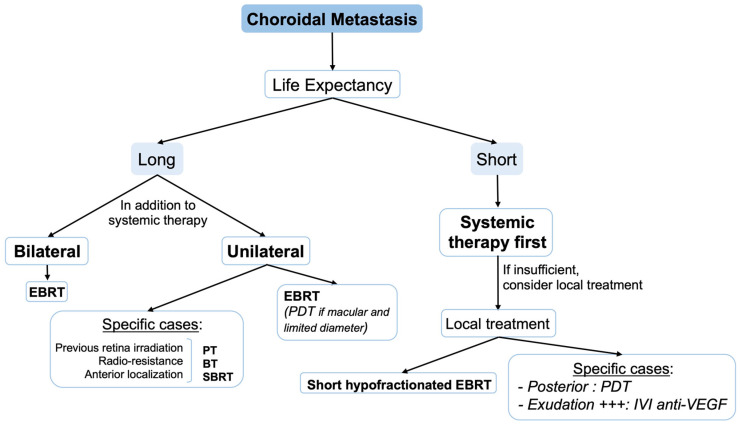
Treatment algorithm for choroidal metastasis. BT: brachytherapy; EBRT: external beam radiotherapy; IVI: intravitreal injection; PDT: photodynamic therapy; PT: proton therapy; SBRT: stereotactic body radiotherapy; VEGF: vascular endothelial growth factor.

Conversely, in patients with long-life expectancy, a more durable and effective treatment is preferred. Conventional EBRT remains the gold standard for such patients, however, PT, SBRT and plaque brachytherapy can be used in specific cases, mostly for unifocal peripheral tumors when the irradiation of the whole posterior pole is not desirable. For macular or juxtapapillary tumors, radiotherapy can be performed but PDT can also be used, with the advantage of preserving adjacent structures (Figure 2).

### 7.1. In Case of Radioresistant Primary Tumor

Some primary tumors require a higher dose of radiotherapy to ensure a complete response. This is the case for melanoma, sarcoma, or renal cancer. It is assumed that metastases from such tumors have the same response profile to radiotherapy and should be treated with a higher dose. The challenge is to deliver this higher dose to the choroidal tumor while minimizing spillover to adjacent tissues. Conventional EBRT is therefore not recommended for this indication and other, better targeted radiation techniques such as ocular PT, plaque brachytherapy or SBRT should be preferred and have shown good results [57].

### 7.2. In Case of Synchronous Ocular and Cerebral Metastases

Choroidal metastases frequently occur in a multimetastatic context and concurrent cerebral metastases are a common finding [67,68,69]. A brain magnetic resonance imaging (MRI) is usually indicated after the discovery of choroidal metastasis (especially in cancers that are prone to cerebral metastases such as lung cancer) and allows for the detection of concomitant brain metastases before treatment. If brain metastases are detected, whole brain irradiation by conventional EBRT is indicated, and can include posterior orbits in the same session. A response is generally observed within a few weeks, in contrast to systemic treatments which are slowed down by the blood-brain barrier.

### 7.3. In Case of Relapse of Ocular Metastasis

When choroidal metastases relapse after conventional EBRT, a second irradiation is generally not possible as the total dose received would exceed the toxic threshold. A more focal radiation technique can be used (plaque brachytherapy, PT, or SBRT) in order to deliver a higher dose in a smaller target volume, sparing surrounding healthy tissues.

### 7.4. In Case of Bilaterality of Ocular Metastases

When choroidal metastases are bilateral (up to 30% in case of breast cancer), conventional EBRT is generally preferred to treat both eyes simultaneously. The question arises of systematically and prophylactically treating the contralateral eye when there is aunilateral choroidal metastasis. Some authors recommend including both choroids in the irradiation field, arguing that the frequency of bilateral metastases is high. Secondary irradiation of the untreated eye might in fact be complex and needs to account for the exit dose received during the initial treatment [70,71]. The evidence supporting the prophylactic irradiation of a healthy eye is not very strong, and some studies suggest that the unintended dose received by the contralateral eye (in order of half the prescription dose using a unilateral beam) is sufficient to eradicate a microscopic disease [72].

### 7.5. In Case of Asymptomatic Ocular Metastases

Asymptomatic choroidal metastasis is usually diagnosed during a routine ocular examination without any functional complaint from the patient. For such tumors, strict monthly examination is necessary to rapidly identify tumor progression. No treatment is generally administered in the absence of ocular complaints; however, minimally invasive techniques such as PDT can be proposed to treat a tumor outside the posterior pole.

## 8. Metastases in Other Ocular Localizations

### 8.1. Iris and Ciliary Body Metastases

Iris and ciliary body are part of the uveal tract as well as the choroid. Metastasis to these tissues is generally asymptomatic (or underreported) in patients under palliative care for multivisceral metastatic spread. When present, pain and blurred vision were the most common symptoms and were related to intraocular inflammation (iritis) or secondary glaucoma [10]. The mechanisms by which the metastases induce ocular hypertension include the obstruction of anterior chamber by the neoplasm itself, the occlusion by cells shed from metastasis in the trabecular meshwork, and neovascular glaucoma. The life expectancy is usually poor in case of iris and ciliary body melanoma, and local treatment is usually withheld, especially if ocular symptoms are absent or mild. Sometimes, local treatment is considered when the patient’s response to systemic therapy is insufficient, or when prompted by the metastases localization or progression. For the latter, targeted radiotherapy could be proposed when tumor is unique and limited to a specific eye quadrant [10]. If multiple masses are present or if the border is poorly limited, EBRT represents another option. Finally, palliative eye care also includes office-based treatment such as anti-VEGF injections if the primary tumor is VEGF-dependent [73].

### 8.2. Retina, Vitreous and Optic Disc Metastases

The recent advances in skin melanoma management, especially with the advent of checkpoint inhibitor molecules, have led to an increase in the prevalence of retina and vitreous metastases. In previous series, these localizations represented less than 1% of ocular metastases, but recent case series have reported more cases and questioned on the actual prevalence of such metastases [8,74]. These lesions being still rare, there are no specific guidelines regarding their management. As for other rare localization, systemic therapy remains the mainstay of the treatment but the blood-retinal and blood-aqueousbarriers limit the diffusion of the anti-cancer molecule into the eye [75]. However, due to the relatively short life expectancy, local treatment is generally postponed and should be used only as a palliative measure to maintain vision at an acceptable level. To this aim, intraocular surgery by vitrectomy can be proposed to remove vitreous cells and improve vision. These local treatments should be discussed in a multidisciplinary concertation with the oncologist, radiotherapist and ophthalmologist.

### 8.3. Extraocular Localization

Conjunctiva and orbital metastases are also rarely reported in the literature. In contrast to ocular localization, they are not directly in contact with neurosensitive and radiosensitive organs (except for orbital metastases bordering the optic nerve). Moreover, they are more accessible for a surgical excision if necessary.

## 9. Conclusions

Ocular metastases are an overlooked problem despite the clear impact on patient’s life. With advances in systemic treatments on one hand, and local treatments on the other hand, tumor control and vision preservation can be increasingly efficient. The combination of systemic and local treatments is complex and highly dependent on the presentation of the ocular metastasis(es) and patient‘s prognosis. Before large evidence-based studies are available, the role of multidisciplinary staff meetings should be emphasized to optimize treatment selection and avoid unnecessary delays that could compromise tumor control, visual preservation, quality of life and autonomy, which could translate into lower societal costs.

## Data Availability

Not applicable.

## References

[1] Corrêa Z.M.d.S., Burmann T.G., Freitas A.M., Ramos G.Z., Marcon I.M. (2005). Prevalence of ocular metastasis in patients with known metastatic disease: Preliminary results. Arq. Bras. Oftalmol..

[2] Welt A., Bogner S., Arendt M., Kossow J., Huffziger A., Pohlkamp C., Steiniger H., Becker U., Alashkar F., Kohl M. (2020). Improved Survival in Metastatic Breast Cancer: Results from a 20-Year Study Involving 1033 Women Treated at a Single Comprehensive Cancer Center. J. Cancer Res. Clin. Oncol..

[3] Konstantinidis L., Rospond-Kubiak I., Zeolite I., Heimann H., Groenewald C., Coupland S.E., Damato B. (2014). Management of Patients with Uveal Metastases at the Liverpool Ocular Oncology Centre. Br. J. Ophthalmol..

[4] Scott A.W., Bressler N.M., Ffolkes S., Wittenborn J.S., Jorkasky J. (2016). Public Attitudes About Eye and Vision Health. JAMA Ophthalmol..

[5] Mathis T., Jardel P., Loria O., Delaunay B., Nguyen A.-M., Lanza F., Mosci C., Caujolle J.-P., Kodjikian L., Thariat J. (2019). New Concepts in the Diagnosis and Management of Choroidal Metastases. Prog. Retin. Eye Res..

[6] Konstantinidis L., Damato B. (2017). Intraocular Metastases—A Review. Asia Pac. J. Ophthalmol. (Phila).

[7] Shields C.L., Shields J.A., Gross N.E., Schwartz G.P., Lally S.E. (1997). Survey of 520 Eyes with Uveal Metastases. Ophthalmology.

[8] Gascon P., Matet A., Gualino V., Denis D., Nguyen A.-M., Papegaey M., Levy N., Arnould T., Kodjikian L., Mathis T. (2022). CLINICAL FEATURES OF RETINAL METASTASES: New Cases Integrated in a Systematic Review of the Literature. Retina.

[9] Shields J.A., Shields C.L., Singh A.D. (2000). Metastatic Neoplasms in the Optic Disc: The 1999 Bjerrum Lecture: Part 2. Arch. Ophthalmol..

[10] Shields C.L., Kaliki S., Crabtree G.S., Peshtani A., Morton S., Anand R.A., Coco G., Shields J.A. (2015). Iris Metastasis from Systemic Cancer in 104 Patients: The 2014 Jerry A. Shields Lecture. Cornea.

[11] Bloch R.S., Gartner S. (1971). The Incidence of Ocular Metastatic Carcinoma. Arch. Ophthalmol..

[12] Eliassi-Rad B., Albert D.M., Green W.R. (1996). Frequency of Ocular Metastases in Patients Dying of Cancer in Eye Bank Populations. Br. J. Ophthalmol..

[13] Nelson C.C., Hertzberg B.S., Klintworth G.K. (1983). A Histopathologic Study of 716 Unselected Eyes in Patients with Cancer at the Time of Death. Am. J. Ophthalmol..

[14] Barak A., Neudorfer M., Heilweil G., Merimsky O., Lowenstein A., Inbar M., Yaal-Hahoshen N. (2007). Decreased Prevalence of Asymptomatic Choroidal Metastasis in Disseminated Breast and Lung Cancer: Argument against Screening. Br. J. Ophthalmol..

[15] Kreusel K.-M., Wiegel T., Stange M., Bornfeld N., Hinkelbein W., Foerster M.H. (2002). Choroidal Metastasis in Disseminated Lung Cancer: Frequency and Risk Factors. Am. J. Ophthalmol..

[16] Su H.-T., Chen Y.-M., Perng R.-P. (2008). Symptomatic Ocular Metastases in Lung Cancer. Respirology.

[17] Wiegel T., Kreusel K.M., Bornfeld N., Bottke D., Stange M., Foerster M.H., Hinkelbein W. (1998). Frequency of Asymptomatic Choroidal Metastasis in Patients with Disseminated Breast Cancer: Results of a Prospective Screening Programme. Br. J. Ophthalmol..

[18] Ferry A.P., Font R.L. (1974). Carcinoma Metastatic to the Eye and Orbit. I. A Clinicopathologic Study of 227 Cases. Arch. Ophthalmol..

[19] Demirci H., Shields C.L., Chao A.-N., Shields J.A. (2003). Uveal Metastasis from Breast Cancer in 264 Patients. Am. J. Ophthalmol..

[20] Shah S.U., Mashayekhi A., Shields C.L., Walia H.S., Hubbard G.B., Zhang J., Shields J.A. (2014). Uveal Metastasis from Lung Cancer: Clinical Features, Treatment, and Outcome in 194 Patients. Ophthalmology.

[21] Hellman S., Weichselbaum R.R. (1995). Oligometastases. J. Clin. Oncol..

[22] Manquez M.E., Brown M.M., Shields C.L., Shields J.A. (2006). Management of Choroidal Metastases from Breast Carcinomas Using Aromatase Inhibitors. Curr. Opin. Ophthalmol..

[23] Yang C.-J., Tsai Y.-M., Tsai M.-J., Chang H.-L., Huang M.-S. (2014). The Effect of Chemotherapy with Cisplatin and Pemetrexed for Choroidal Metastasis of Non-Squamous Cell Carcinoma. Cancer Chemother. Pharmacol..

[24] Cui Z.-H., Zhang Y., Liang L.-L., Li Z.-H., Abramova I., Hao Q. (2017). Development of a New Choroidal Metastasis in Resistance to Crizotinib Therapy in Anaplastic Lymphoma Kinase-Rearranged Non-Small Cell Lung Cancer. Int. J. Ophthalmol..

[25] Okuma Y., Tanaka Y., Kamei T., Hosomi Y., Okamura T. (2015). Alectinib for Choroidal Metastasis in a Patient with Crizotinib-Resistant ALK Rearranged Positive Non-Small Cell Lung Cancer. Onco Targets Ther..

[26] Bill A., Törnquist P., Alm A. (1980). Permeability of the Intraocular Blood Vessels. Trans. Ophthalmol. Soc. U. K..

[27] Fu Y., Xiao W., Mao Y. (2022). Recent Advances and Challenges in Uveal Melanoma Immunotherapy. Cancers.

[28] Tao T., Liu Y., Zhang J., Huang L., Tao Y. (2022). Dynamic Observation: Immune-Privileged Microenvironment Limited the Effectiveness of Immunotherapy in an Intraocular Metastasis Mouse Model. Ophthalmic Res..

[29] Li X., Liang Y., Wang J., Hu Z., Yuan Z.-L., Xie P. (2021). Pembrolizumab for Choroidal Metastases from Non-Small Cell Lung Cancer: A Case Report and Literature Review. Hum. Vaccin. Immunother..

[30] Cortes J., Rugo H.S., Cescon D.W., Im S.-A., Yusof M.M., Gallardo C., Lipatov O., Barrios C.H., Perez-Garcia J., Iwata H. (2022). Pembrolizumab plus Chemotherapy in Advanced Triple-Negative Breast Cancer. N. Engl. J. Med..

[31] Garon E.B., Rizvi N.A., Hui R., Leighl N., Balmanoukian A.S., Eder J.P., Patnaik A., Aggarwal C., Gubens M., Horn L. (2015). Pembrolizumab for the Treatment of Non-Small-Cell Lung Cancer. N. Engl. J. Med..

[32] Motzer R.J., Escudier B., McDermott D.F., George S., Hammers H.J., Srinivas S., Tykodi S.S., Sosman J.A., Procopio G., Plimack E.R. (2015). Nivolumab versus Everolimus in Advanced Renal-Cell Carcinoma. N. Engl. J. Med..

[33] Paz-Ares L., Luft A., Vicente D., Tafreshi A., Gümüş M., Mazières J., Hermes B., Çay Şenler F., Csőszi T., Fülöp A. (2018). Pembrolizumab plus Chemotherapy for Squamous Non-Small-Cell Lung Cancer. N. Engl. J. Med..

[34] Haslam A., Prasad V. (2019). Estimation of the Percentage of US Patients With Cancer Who Are Eligible for and Respond to Checkpoint Inhibitor Immunotherapy Drugs. JAMA Netw. Open.

[35] Larkin J., Chiarion-Sileni V., Gonzalez R., Grob J.-J., Rutkowski P., Lao C.D., Cowey C.L., Schadendorf D., Wagstaff J., Dummer R. (2019). Five-Year Survival with Combined Nivolumab and Ipilimumab in Advanced Melanoma. N. Engl. J. Med..

[36] Maller B., Salvatori S., Tanvetyanon T. (2022). Outcomes of Intraocular Metastasis From Lung Cancer in the Era of Targeted Therapy: A Systematic Review and Pooled Analysis. Clin. Lung Cancer.

[37] Parrozzani R., Frizziero L., Testi I., Miglionico G., Perrini P., Pulze S., Pilotto E., Midena E. (2016). Intraocular Metastases Secondary to Breast Carcinoma Correlates with Upregulation of Estrogen and Progesterone Receptor Expression in the Primary Tumor. Investig. Ophthalmol. Vis. Sci..

[38] Hortobagyi G.N., Stemmer S.M., Burris H.A., Yap Y.-S., Sonke G.S., Paluch-Shimon S., Campone M., Blackwell K.L., André F., Winer E.P. (2016). Ribociclib as First-Line Therapy for HR-Positive, Advanced Breast Cancer. N. Engl. J. Med..

[39] Parakh S., Das S., Maheshwari S., Gupta V., Luthra G., Luthra S. (2022). Regression of Choroidal Metastasis from Breast Carcinoma with Palbociclib. Int. J. Retin. Vitr..

[40] Lin N.U., Borges V., Anders C., Murthy R.K., Paplomata E., Hamilton E., Hurvitz S., Loi S., Okines A., Abramson V. (2020). Intracranial Efficacy and Survival With Tucatinib Plus Trastuzumab and Capecitabine for Previously Treated HER2-Positive Breast Cancer With Brain Metastases in the HER2CLIMB Trial. J. Clin. Oncol..

[41] Bartsch R., Berghoff A.S., Furtner J., Marhold M., Bergen E.S., Roider-Schur S., Starzer A.M., Forstner H., Rottenmanner B., Dieckmann K. (2022). Trastuzumab Deruxtecan in HER2-Positive Breast Cancer with Brain Metastases: A Single-Arm, Phase 2 Trial. Nat. Med..

[42] Chen F., Chen N., Lv Z., Li L., Cui J. (2021). Efficacy of Second-Line Treatments for Patients with Advanced Human Epidermal Growth Factor Receptor 2 Positive Breast Cancer after Trastuzumab-Based Treatment: A Systematic Review and Bayesian Network Analysis. J. Cancer.

[43] Vickers A.D., Winfree K.B., Cuyun Carter G., Kiiskinen U., Jen M.-H., Stull D., Kaye J.A., Carbone D.P. (2019). Relative Efficacy of Interventions in the Treatment of Second-Line Non-Small Cell Lung Cancer: A Systematic Review and Network Meta-Analysis. BMC Cancer.

[44] El Karak F., Gh Haddad F., Eid R., Al Ghor M., El Rassy E., Ahmadieh N., Choullamy T., Halim N.A., Tfayli A., Farhat F. (2019). Lung Cancer and Immunotherapy: A Real-Life Experience from Second Line and Beyond. Future Oncol..

[45] Dougherty T.J., Gomer C.J., Henderson B.W., Jori G., Kessel D., Korbelik M., Moan J., Peng Q. (1998). Photodynamic Therapy. J. Natl. Cancer Inst..

[46] Gragoudas E.S., Egan K.M., Saornil M.A., Walsh S.M., Albert D.M., Seddon J.M. (1993). The Time Course of Irradiation Changes in Proton Beam-Treated Uveal Melanomas. Ophthalmology.

[47] Rudoler S.B., Corn B.W., Shields C.L., De Potter P., Hyslop T., Shields J.A., Curran W.J. (1997). External Beam Irradiation for Choroid Metastases: Identification of Factors Predisposing to Long-Term Sequelae. Int. J. Radiat. Oncol. Biol. Phys..

[48] Rudoler S.B., Shields C.L., Corn B.W., De Potter P., Hyslop T., Curran W.J., Shields J.A. (1997). Functional Vision Is Improved in the Majority of Patients Treated with External-Beam Radiotherapy for Choroid Metastases: A Multivariate Analysis of 188 Patients. J. Clin. Oncol..

[49] d’Abbadie I., Arriagada R., Spielmann M., Lê M.G. (2003). Choroid Metastases: Clinical Features and Treatments in 123 Patients. Cancer.

[50] Shields C.L., Shields J.A., De Potter P., Quaranta M., Freire J., Brady L.W., Barrett J. (1997). Plaque Radiotherapy for the Management of Uveal Metastasis. Arch. Ophthalmol..

[51] Perri P., Chiarelli M., Monari P., Ravalli L., Mazzeo V. (1992). Choroidal Metastases. Echographic Experience from 42 Patients. Acta Ophthalmol..

[52] Kamran S.C., Collier J.M., Lane A.M., Kim I., Niemierko A., Chen Y.-L.E., MacDonald S.M., Munzenrider J.E., Gragoudas E., Shih H.A. (2014). Outcomes of Proton Therapy for the Treatment of Uveal Metastases. Int. J. Radiat. Oncol. Biol. Phys..

[53] Tsina E.K., Lane A.M., Zacks D.N., Munzenrider J.E., Collier J.M., Gragoudas E.S. (2005). Treatment of Metastatic Tumors of the Choroid with Proton Beam Irradiation. Ophthalmology.

[54] Gragoudas E.S., Carroll J.M. (1979). Multiple Choroidal Metastasis from Bronchial Carcinoid Treated with Photocoagulation and Proton Beam Irradiation. Am. J. Ophthalmol..

[55] Bellmann C., Fuss M., Holz F.G., Debus J., Rohrschneider K., Völcker H.E., Wannenmacher M. (2000). Stereotactic Radiation Therapy for Malignant Choroidal Tumors: Preliminary, Short-Term Results. Ophthalmology.

[56] Cho K.R., Lee K.M., Han G., Kang S.W., Lee J.-I. (2018). Gamma Knife Radiosurgery for Cancer Metastasized to the Ocular Choroid. J. Korean Neurosurg. Soc..

[57] Mathis T., Caujolle J.-P., Thariat J. (2020). Choroidal Metastasis From Melanoma Treated by Cyberknife Irradiation. JAMA Ophthalmol..

[58] Ares W.J., Tonetti D., Yu J.Y., Monaco E.A., Flickinger J.C., Lunsford L.D. (2017). Gamma Knife Radiosurgery for Uveal Metastases: Report of Three Cases and a Review of the Literature. Am. J. Ophthalmol..

[59] Mathis T., Maschi C., Mosci C., Espensen C.A., Rosier L., Favard C., Tick S., Remignon C.-H., Ligorio P., Bonin N. (2021). Comparative effectiveness of proton beam versus photodynamic therapy to spare the vision in circumscribed choroidal hemangioma. Retina.

[60] Ghodasra D.H., Demirci H. (2016). Photodynamic Therapy for Choroidal Metastasis. Am. J. Ophthalmol..

[61] Kaliki S., Shields C.L., Al-Dahmash S.A., Mashayekhi A., Shields J.A. (2012). Photodynamic Therapy for Choroidal Metastasis in 8 Cases. Ophthalmology.

[62] Hua R., Li W., Wu W., Tao J., Peng Q. (2017). Failure of Ocular Photodynamic Therapy for Secondary Choroidal Metastasis: A Case Report and Literature Review. Oncotarget.

[63] Langley R.R., Fidler I.J. (2011). The Seed and Soil Hypothesis Revisited--the Role of Tumor-Stroma Interactions in Metastasis to Different Organs. Int. J. Cancer.

[64] Johnstone S., Logan R.M. (2007). Expression of Vascular Endothelial Growth Factor (VEGF) in Normal Oral Mucosa, Oral Dysplasia and Oral Squamous Cell Carcinoma. Int. J. Oral Maxillofac. Surg..

[65] Lin C.-J., Tsai Y.-Y. (2015). The Effect of Intravitreal Bevacizumab and Transpupillary Thermotherapy on Choroidal Metastases and Literature Review. Indian J. Ophthalmol..

[66] Fenicia V., Abdolrahimzadeh S., Mannino G., Verrilli S., Balestrieri M., Recupero S.M. (2014). Intravitreal Bevacizumab in the Successful Management of Choroidal Metastases Secondary to Lung and Breast Cancer Unresponsive to Systemic Therapy: A Case Series. Eye.

[67] Jardel P., Sauerwein W., Olivier T., Bensoussan E., Maschi C., Lanza F., Mosci C., Gastaud L., Angellier G., Marcy P.-Y. (2014). Management of Choroidal Metastases. Cancer Treat. Rev..

[68] Amer R., Pe’er J., Chowers I., Anteby I. (2004). Treatment Options in the Management of Choroidal Metastases. Ophthalmologica.

[69] Kreusel K.-M., Bechrakis N.E., Wiegel T., Krause L., Foerster M.H. (2008). Incidence and Clinical Characteristics of Symptomatic Choroidal Metastasis from Lung Cancer. Acta Ophthalmol..

[70] Rosset A., Zografos L., Coucke P., Monney M., Mirimanoff R.O. (1998). Radiotherapy of Choroidal Metastases. Radiother. Oncol..

[71] Wiegel T., Kreusel K.M., Schmidt S., Bornfeld N., Foerster M.H., Hinkelbein W. (1999). Radiotherapy of Unilateral Choroidal Metastasis: Unilateral Irradiation or Bilateral Irradiation for Sterilization of Suspected Contralateral Disease?. Radiother. Oncol..

[72] Wiegel T., Bottke D., Kreusel K.-M., Schmidt S., Bornfeld N., Foerster M.H., Hinkelbein W. (2002). German Cancer Society External Beam Radiotherapy of Choroidal Metastases—Final Results of a Prospective Study of the German Cancer Society (ARO 95-08). Radiother. Oncol..

[73] Wong M., Lee W.B., Halpern R.L., Frank J.H. (2017). Ciliary Body Metastasis from Renal Cell Carcinoma Successfully Treated with Intravitreal Bevacizumab. Am. J. Ophthalmol. Case Rep..

[74] Francis J.H., Berry D., Abramson D.H., Barker C.A., Bergstrom C., Demirci H., Engelbert M., Grossniklaus H., Hubbard B., Iacob C.E. (2020). Intravitreous Cutaneous Metastatic Melanoma in the Era of Checkpoint Inhibition: Unmasking and Masquerading. Ophthalmology.

[75] Del Amo E.M., Rimpelä A.-K., Heikkinen E., Kari O.K., Ramsay E., Lajunen T., Schmitt M., Pelkonen L., Bhattacharya M., Richardson D. (2017). Pharmacokinetic Aspects of Retinal Drug Delivery. Prog. Retin. Eye Res..

